# Performance Characteristics of the Vidas SARS-CoV-2 IgM and IgG Serological Assays

**DOI:** 10.1128/JCM.02292-20

**Published:** 2021-03-19

**Authors:** Nathalie Renard, Soizic Daniel, Nadège Cayet, Matthieu Pecquet, Frédérique Raymond, Sylvie Pons, Julien Lupo, Carole Tourneur, Catherine Pretis, Guillaume Gerez, Patrick Blasco, Maxime Combe, Imen Canova, Mylène Lesénéchal, Franck Berthier

**Affiliations:** a R&D bioMérieux, Marcy l’Etoile, France; b Laboratoire de Biologie, Centre Hospitalier Saint Joseph Saint Luc, Lyon, France; c Laboratoire Commun de Recherche Hospices Civils de Lyon-bioMérieux, Centre Hospitalier Lyon-Sud, Pierre-Bénite, France; d Institut de Biologie Structurale (IBS), CEA, CNRS, Université Grenoble-Alpes; Laboratoire de Virologie, Centre Hospitalier Universitaire Grenoble-Alpes, Grenoble, France; Cepheid

**Keywords:** COVID-19, SARS-CoV-2, Vidas, diagnosis, immunoserology

## Abstract

The COVID-19 pandemic, caused by the new severe acute respiratory syndrome coronavirus 2 (SARS-CoV-2), continues to spread worldwide. Serological testing for SARS-CoV-2-specific antibodies plays an important role in understanding and controlling the pandemic, notably through epidemiological surveillance. Well-validated and highly specific SARS-CoV-2 serological assays are urgently needed. We describe here the analytical and clinical performance of Vidas SARS-CoV-2 IgM and Vidas SARS-CoV-2 IgG, two CE-marked, emergency use authorization (EUA)-authorized, automated, qualitative assays for the detection of SARS-CoV-2-specific IgM and IgG, respectively. Both assays showed high within-run and within-laboratory precision (coefficients of variation < 11.0%) and very low cross-reactivity toward sera of patients with a past common coronavirus or respiratory virus infection. Clinical specificity determined on up to 989 prepandemic healthy donors was ≥99% with a narrow 95% confidence interval for both IgM and IgG assays. Clinical sensitivity was determined on up to 232 samples from 130 reverse transcriptase PCR (RT-PCR)-confirmed SARS-CoV-2 patients. The positive percent agreement (PPA) with SARS-CoV-2 PCR reached 100% at ≥16 days (Vidas SARS-CoV-2 IgM) and ≥32 days (Vidas SARS-CoV-2 IgG) of symptom onset. Combined IgM/IgG test results improved the PPA compared to each test alone. SARS-CoV-2 IgG seroconversion followed closely that of SARS-CoV-2 IgM and remained stable over time, while SARS-CoV-2 IgM levels rapidly declined. Interestingly, SARS-CoV-2-specific IgM and IgG responses were significantly higher in COVID-19 hospitalized versus nonhospitalized patients. Altogether, the Vidas SARS-CoV-2 IgM and IgG assays are highly specific and sensitive serological tests suitable for the reliable detection of past acute SARS-CoV-2 infections.

## Abstract (precis)

The COVID-19 pandemic, caused by the new severe acute respiratory syndrome coronavirus 2 (SARS-CoV-2), continues to spread worldwide. Serological testing for SARS-CoV-2-specific antibodies plays an important role in understanding and controlling the pandemic, notably through epidemiological surveillance.

## INTRODUCTION

Coronavirus disease 19 (COVID-19) is an infectious disease caused by the newly discovered severe acute respiratory syndrome coronavirus 2 (SARS-CoV-2) (1, 2). Within 3 months of its emergence in China in December 2019, COVID-19 has been declared a global pandemic by the World Health Organization (WHO). As of 29 October 2020, nearly 45 million COVID-19 cases and 1.2 million deaths had been reported worldwide (3–5). Accurate diagnosis is essential in managing the pandemic, not only to identify, isolate, and treat affected patients, but also to characterize the epidemiology of virus transmission and develop national and international surveillance programs. WHO recommends molecular testing of SARS-CoV-2 nucleic acids for acute-phase diagnosis of suspected cases (6–8). Several nucleic acid amplification tests (NAATs), mostly based on quantitative reverse transcriptase PCR (RT-qPCR), have received the Conformité Européenne (CE) mark and have been approved by the U.S. Food and Drug Administration (FDA) under emergency use authorization (EUA) (9–11). On the other hand, serological testing for SARS-CoV-2-specific antibodies, especially immunoglobulin M (IgM) and immunoglobulin G (IgG), is not recommended as the primary method for the diagnosis of acute cases. It plays, however, an essential role in the diagnosis of past SARS-CoV-2 infection and in ongoing immunological and epidemiological surveillance. Serological testing might also complement molecular testing to confirm suspected cases not detected by molecular assays, either due to late (>7 days after infection) or improper sample collection. Finally, serology screening may allow the identification of convalescent plasma donors for use as potential therapy against COVID-19 (9–18).

SARS-CoV-2 serological testing is facing several challenges. Among them, sensitivity and specificity should be well defined for the target population and validated at different postinfection time windows. Specificity is particularly critical in the current pandemic phase, as seroprevalence in the population is still low. In such low-incidence settings, a specificity of >99% and a narrow 95% confidence interval (95% CI) are required to ensure a high positive predictive value (PPV) (11, 19, 20). Accordingly, the antigens used to design serology tests should be properly selected, and cross-reactivity with antibodies directed against other antigens, including from other coronaviruses, should be verified. A huge number of serology assays have been developed and marketed in the last few months, 56 of which received the FDA’s EUA (as of 29 October 2020) (21, 22). Clinical performance data of commercial tests are still scant, and examples of poorly performing tests have even been reported (9). Therefore, there is an urgent need for well-validated and performant serology tests, notably demonstrating very high specificity.

We describe here the analytical and clinical performance of Vidas SARS-CoV-2 IgM and Vidas SARS-CoV-2 IgG, two CE-marked and EUA-authorized automated qualitative assays for the detection of SARS-CoV-2-specific IgM and IgG, respectively, in serum or plasma. The kinetics of SARS-CoV-2-specific IgM and IgG seroconversion using the Vidas SARS-CoV-2 IgM and Vidas SARS-CoV-2 IgG assays were also compared in hospitalized and nonhospitalized COVID-19 patients.

## MATERIALS AND METHODS

### Patients and samples.

SARS-CoV-2-positive samples were collected after approval by the Ethics Committee Review Committee Board (RCB) 2020-A00932-37. Informed consent was obtained in accordance with local regulations. Prepandemic samples (from healthy subjects and from donors with other medical conditions) were collected in accordance with the Declaration of Helsinki, as revised in 2013. Collected sera and plasma were stored frozen (<−20°C) until further testing.

Serum from up to 989 healthy prepandemic adult donors collected before September 2019 at two geographical sites (Etablissement Français du Sang [EFS], France; Clinilabs, Inc., United States) was used to determine the assay specificity of the Vidas SARS-CoV-2 IgM, IgG, and combined IgM/IgG tests (defined as negative if both Vidas SARS-CoV-2 IgM and IgG assays are negative).

**FIG 1:**
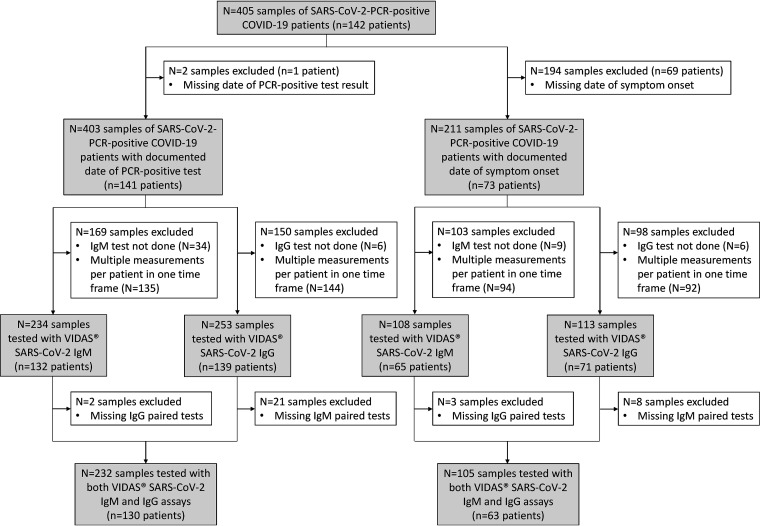
Study flow diagram. Description of SARS-CoV-2-positive samples used to determine the positive percent agreement relative to the time of RT-PCR positive test result and to the time of symptom onset. The number of and reason for sample exclusion are indicated in the white boxes. Included samples are indicated in the gray boxes. Altogether, out of the 405 collected samples (from 142 SARS-CoV-2-positive patients), 232 samples from 130 patients with a documented date for the SARS-CoV-2-specific PCR positive test and 105 samples from 63 patients with a documented date of symptom onset were tested with both the Vidas SARS-CoV-2 IgM and IgG assays (paired tests).

For the evaluation of the positive percent agreement (PPA), 405 serum or plasma samples from 142 symptomatic patients (60 hospitalized, 61 nonhospitalized, 21 of unknown hospitalization status) diagnosed with COVID-19 and confirmed positive for SARS-CoV-2 by molecular testing (cobas SARS-CoV-2, Roche 09175431190, or real-time reverse transcriptase [RT-PCR] assays for the detection of SARS-CoV-2, Institut Pasteur, Paris [23]; performed at the collection site) (Fig. 1) were collected at three local hospitals (Centre Hospitalier Saint Joseph Saint Luc, Lyon, France; Centre de Ressources Biologiques [CRB] des Hospices Civils de Lyon, CRB Nord and CRB Sud, Lyon, France) between March 31 and June 2, 2020. Samples were tested with the Vidas SARS-CoV-2 IgM and IgG assays, and paired measurements were considered for the combined IgM/IgG test results (defined as positive if at least one of the Vidas SARS-CoV-2 IgM and/or IgG assays is positive) (Fig. 1). The PPA was evaluated according to weekly time frames (0 to 7, 8 to 15, 16 to 23, 24 to 31, or ≥32 days) relative to the time from an RT-PCR positive result and from symptom onset, when documented (Fig. 1).

For the evaluation of serum cross-reactivity, up to 276 frozen prepandemic sera (i.e., negative for SARS-CoV-2) collected from patients with other potentially interfering infections or medical conditions (bioMérieux, Centre Hospitalier Universitaire Grenoble-Alpes, and Saint Joseph Saint Luc Lyon collections) were tested with the Vidas SARS-CoV-2 IgM (276 sera from 33 medical conditions; 1 to 30 sera per condition) and the Vidas SARS-CoV-2 IgG (261 sera from 33 medical conditions; 2 to 30 sera per condition) assays.

### Serological assays.

Vidas SARS-CoV-2 IgM (423833) and Vidas SARS-CoV-2 IgG (423834) (bioMérieux, France) are automated qualitative CE-*in vitro* diagnostic (IVD) assays developed for the Vidas family of instruments and based on a two-step enzyme immunoassay combined with an enzyme-linked fluorescent assay (ELFA) detection technique. The Vidas SARS-CoV-2 IgM and Vidas SARS-CoV-2 IgG assays are intended for use as an aid to determine if individuals may have been exposed to and infected by SARS-CoV-2 and if they have mounted a specific anti-SARS-CoV-2 IgM and IgG immune response. These assays allow the detection of SARS-CoV-2-specific IgM and IgG, respectively, from 100 μl serum or plasma (lithium heparin). The Vidas SARS-CoV-2 IgM and Vidas SARS-CoV-2 IgG serological assays were conducted according to the manufacturer’s instructions. Briefly, a solid-phase receptacle coated with the antigen (recombinant SARS-CoV-2 receptor-binding domain [RBD] of the viral spike protein) serves as both solid-phase and pipetting device. After the sample dilution step, SARS-CoV-2-specific IgM and IgG are captured on the coated antigen, and unbound components are washed out. In the second step, human IgM (Vidas SARS-CoV-2 IgM) or IgG (Vidas SARS-CoV-2 IgG) are specifically detected by mouse monoclonal antibodies conjugated to alkaline phosphatase and directed against human IgM or IgG, respectively. Unbound components are eliminated by washing, and detection is performed by incubation with the substrate (4-methyl-umbelliferyl phosphate) followed by measurement of the fluorescent product (4-methyl-umbelliferone) at 450 nm. A relative fluorescence value (RFV) is generated (background reading subtracted from the final fluorescence reading). The assay is conducted with a standard (S1) and a positive control (C1) that contains humanized recombinant anti-SARS-CoV-2 antibody, either IgM or IgG depending on the assay. A negative control (C2) is also supplied. The results are automatically calculated by the instrument, according to the S1 standard, and an index value (*i*) is obtained (where *i* = RFV_sample_/RFV_S1_). The test is interpreted as negative when *i* < 1.00 and positive when *i* ≥ 1.00. The positivity cutoff values for the IgM and IgG tests were determined from a healthy prepandemic cohort (259 [IgM test] and 120 [IgG test] samples collected prior to August 2019), using nonparametric 99th percentile because of normality rejection for the IgM positivity cutoff and using the (99) tolerance intervals approach after Box-Cox transformation (24) for the IgG positivity cutoff (99th percentile index values at a 99% confidence level) (data not shown).

### Statistical analysis.

Assay precision was evaluated according to the Clinical and Laboratory Standards Institute (CLSI) EP05-A3 guideline (25) using the variance component method and restricted maximum likelihood (REML) using the SAS Enterprise Guide 7.13 HF8 software.

Specificity and sensitivity (PPA) estimates were evaluated according to the CLSI EP12-A2 guideline (26). The 95% confidence intervals (95% CI) were computed (either as score confidence interval if the specificity or sensitivity [PPA] was in the range ]5%, 95%[ or as exact confidence interval otherwise) using the SAS Enterprise Guide 7.13 HF8 software.

PPA was evaluated per time windows (in days) relative to the day of RT-PCR positive result and of symptom onset (when documented). To avoid a statistical bias, only one patient’s measurement per time period was included in the analysis. In case of multiple patient’s measurements in one period, the first available measurement was considered for the calculation. Therefore, depending on the total number of longitudinal tests performed, each patient contributed with one to five test results in the five time windows considered (0 to 7, 8 to 15, 16 to 23, 24 to 31, and ≥32 days).

The positive predictive value (PPV) and the negative predictive value (NPV) were calculated assuming a prevalence of 5%, as recommended by the FDA for the EUA application (22), and the respective 95% CI were computed according to Mercaldo et al. (27) using the SAS Enterprise Guide 7.13 HF8 software.

Vidas SARS-CoV-2 index values were displayed per time frame as Tukey box plots. Two-group comparisons of index values per time frame between hospitalized and nonhospitalized patients were performed using the nonparametric two-tailed Mann-Whitney *U* test (MWU test) with normal approximation. In case of multiple group comparisons, the Bonferroni method was applied for controlling the 5% overall probability of a false-significant result. Accordingly, for three-group comparisons, *P* values of <0.017 were considered statistically significant.

## RESULTS

### Analytical performance of the Vidas SARS-CoV-2 IgM and IgG assays.

Within-run and within-laboratory precisions of the ELFA-based tests were determined using three samples (one negative and two positive for SARS-CoV-2 IgM and IgG). Samples were run in triplicate on one Vidas instrument, twice a day over 10 days (with an instrument calibration every second day), using one assay lot, thus generating 60 measurement values per sample. The coefficient of variation (%CV) for repeatability (within-run precision) did not exceed 9.3% and 5.9% for the Vidas SARS-CoV-2 IgM and IgG assays, respectively. The %CV for within-laboratory precision was also low, reaching a maximum of 10.7% and 6.9% for the Vidas SARS-CoV-2 IgM and IgG assays, respectively (see Table S1 in the supplemental material).

Analytical specificity and sensitivity of the Vidas SARS-CoV-2 IgM and IgG assays were verified through various experiments. First, we ruled out a possible cross-reactivity of the anti-human-IgM (Vidas SARS-CoV-2 IgM) or of the anti-human-IgG (Vidas SARS-CoV-2 IgG) with human IgG or IgM, respectively, which might produce false-positive results. Spike-in experiments in negative samples using either human recombinant monoclonal anti-SARS-CoV-2 IgG (10 μg/ml) in the Vidas SARS-CoV-2 IgM assay or human recombinant monoclonal anti-SARS-CoV-2 IgM (3 μg/ml) in the Vidas SARS-CoV-2 IgG assay demonstrated neither reactivity of the alkaline-phosphatase-conjugated anti-human-IgM toward human IgG nor reactivity of the anti-human-IgG toward human IgM (*n* = 10; data not shown). Second, we ruled out a possible competition between anti-SARS-CoV-2 IgM and IgG for binding to the coated SARS-CoV-2 antigen, which might interfere with the respective assays and generate false-negative results. Spike-in experiments in positive samples (with index values ranging from 1.8 to 20.6) using an excess of human recombinant monoclonal anti-SARS-CoV-2 IgG (10 μg/ml) in the Vidas SARS-CoV-2 IgM assay or of human recombinant monoclonal anti-SARS-CoV-2 IgM (3 μg/ml) in the Vidas SARS-CoV-2 IgG assay did not impact the qualitative test results and resulted in a maximum deviation from the respective control of 17.6% (*n* = 12; data not shown). Third, we evaluated the impact of serum inactivation (56°C for 30 min), which might be applied by diagnostics laboratories to inactivate potentially infectious samples (28), on test results of 10 negative and 10 positive samples. Heat inactivation did not impact the qualitative test results of the Vidas SARS-CoV-2 IgM and IgG assays (20/20 = 100% concordance), and the maximum deviation to the (nonheated) control among 18/20 samples was 17%. However, one negative sample (Vidas SARS-CoV-2 IgG) and one positive sample (Vidas SARS-CoV-2 IgM) yielded significantly divergent index values (data not shown). Therefore, heat inactivation of sera prior to Vidas SARS-CoV-2 testing should be preferably avoided. Finally, we evaluated the possible cross-reactivity of components of the assay (SARS-CoV-2 antigen RBD or immunoglobulins) with human sera from patients with other infections (including other coronaviruses) or medical conditions (e.g., rheumatoid factor) (29) that might interfere with the assay and yield false-positive results. Up to 276 (Vidas SARS-CoV-2 IgM) and 261 (Vidas SARS-CoV-2 IgG) sera of SARS-CoV-2-negative patients with other infections or conditions were tested, and the number of positive test results was evaluated (Table 1). None of the 18 sera of patients with a history of infection with the human coronaviruses CoV-NL63, CoV-229E, CoV-HKU1, or CoV-OC43 (genera *Alphacoronavirus* and *Betacoronavirus*) were positive in the Vidas SARS-CoV-2 IgG assay, while the serum of one CoV-NL63-positive patient was positive in the Vidas SARS-CoV-2 IgM assay. Only two out of 261 (0.8%) tested sera were positive in the Vidas SARS-CoV-2 IgG assay. They belonged to an HIV-positive and a respiratory syncytial virus A (RSV A)-positive patient, respectively. On the other hand, 10 out of 276 (3.6%) tested sera were positive in the Vidas SARS-CoV-2 IgM assay. Apart from the one CoV-NL63-positive sample mentioned above, six sera were from patients presenting autoantibodies (antinuclear antibody, rheumatoid factor), two were from patients with a history of parasite infection (Plasmodium falciparum, Trypanosoma cruzi), and one was from a past rhinovirus/enterovirus infection. The index values associated with these 12 cross-reactive sera were low, with a median (interquartile range) of 2.1 (range, 1.4 to 3.8). None of the sera from patients infected with other respiratory viruses, including influenza virus, parainfluenza virus, metapneumovirus, or adenovirus, were reactive.

**TABLE 1:** Cross-reactivity of human sera from patients with other infections or medical conditions potentially interfering with the Vidas SARS-CoV-2 IgM and IgG assays

Sample category	Data for Vidas SARS-CoV-2 IgM:		Data for Vidas SARS-CoV-2 IgG:	
	No. of samples tested	No. of positive tests	No. of samples tested	No. of positive tests
Pregnant women	5	0	5	0
Antinuclear antibody (ANA)^*a*^	47	2	47	0
Rheumatoid factor	19	4	19	0
Human anti-mouse antibody (HAMA)	5	0	5	0
Borrelia burgdorferi^*b*^	10	0	6	0
Haemophilus influenzae *B*	5	0	5	0
Plasmodium falciparum	3	1	3	0
Toxoplasma gondii^*b*^	10	0	6	0
Treponema pallidum	3	0	3	0
Trypanosoma cruzi	5	1	5	0
Hepatitis A virus (HAV)	3	0	3	0
Hepatitis B virus (HBV)	5	0	5	0
Hepatitis C virus (HCV)	5	0	5	0
Hepatitis E virus (HEV)^*b*^	7	0	6	0
Herpes simplex virus (HSV)^*b*^	6	0	6	0
Human immunodeficiency virus (HIV)	5	0	5	1
Cytomegalovirus (CMV)	4	0	3	0
Measles virus (MV)	4	0	3	0
Mumps virus (MuV)	1	0	3	0
Rubella virus (RuV)^*b*^	10	0	6	0
Dengue virus (DENV)	3	0	3	0
West Nile virus (WNV)	4	0	3	0
Yellow fever virus (YFV)	4	0	3	0
Zika virus (ZIKV)^*b*^	5	0	5	0
Adenovirus (AdV)	2	0	2	0
Metapneumovirus (MPV)	4	0	4	0
Rhinovirus/enterovirus (RV/EnteroV)^*c*^	20	1	20	0
Influenza A and B virus (IAV/IBV)	30	0	30	0
Parainfluenza viruses 1/2/3 (PIV-1/2/3)	11	0	11	0
Respiratory syncytial virus A or B (RSV A or B)	13	0	13	1
Coronavirus NL63/HKU1 (CoV-NL63/HKU1)^*d*^	9	1	9	0
Coronavirus 229E (CoV-229E)	7	0	7	0
Coronavirus OC43 (CoV-OC43)	2	0	2	0
Total	276	10	261	2

a Includes anti-DNA, anti-Sjögren's-syndrome-related antigen A (SSA), anti-Sjögren's-syndrome-related antigen B (SSB), and anti-Sm/RNP antibodies.

b The proportion of acute infection (i.e., IgM-positive for the respective infectious agent when IgM levels were characterized) among samples tested with the Vidas SARS-CoV-2 IgM assay was Borrelia burgdorferi, 5/10; Toxoplasma gondii, 5/10; HEV, 3/7; HSV, 3/6; RuV, 5/10; ZIKV, 3/5.

c One out of twenty sera was from a patient with a bocavirus (BoV) coinfection and was negative in both the Vidas SARS-CoV-2 IgM and IgG assays.

d Six out of the nine sera tested were from patients positive for CoV-NL63, and three were from patients positive for CoV-NL63 and/or CoV-HKU1.

### Clinical performance of Vidas SARS-COV-2 IgM and IgG assays.

The clinical specificities of the Vidas SARS-CoV-2 IgM and IgG assays were assessed using sera from up to 989 prepandemic healthy volunteers collected in France and in the United States before September 2019. A total of 308 sera were tested with the Vidas SARS-CoV-2 IgM assay, and 989 sera were tested with the Vidas SARS-CoV-2 IgG assay. The combined IgM/IgG assay specificity (defined as both Vidas SARS-CoV-2 IgM and IgG assays being negative) was determined on 308 paired Vidas SARS-CoV-2 IgM and IgG tests (Tables S2 and S3). A total of 306/308 (Vidas SARS-CoV-2 IgM) and 988/989 (Vidas SARS-CoV-2 IgG) SARS-CoV-2-negative sera were negative, corresponding to a specificity (95% CI) of 99.4% (range, 97.7 to 99.9%) and 99.9% (99.4 to 100%) for the Vidas SARS-CoV-2 IgM and IgG assays, respectively (Table S3). The specificity (95% CI) of the Vidas SARS-CoV-2 IgG test on the common cohort (*n* = 308) was 100.0% (98.8 to 100.0%). The specificity (95% CI) of the combined IgM and IgG serology tests (306/308 tests negative in both assays) was 99.4% (97.7 to 99.9%) (Table S3).

The clinical sensitivity of the Vidas SARS-CoV-2 IgM and IgG assays was assessed using 405 samples collected from 142 patients confirmed positive for SARS-CoV-2 by RT-PCR. The positive percent agreement (PPA) of the serology tests with the RT-PCR test results was calculated per weekly time windows (0 to 7, 8 to 15, 16 to 23, 24 to 31, and ≥32 days) relative to the time from the PCR positive result and to the time from symptom onset. No more than one patient sample per time window was included in the calculation (Fig. 1 and Table 2). The PPA calculated on all available samples for the Vidas SARS-CoV-2 IgM and IgG assays are shown in Tables S4 and S5, respectively. For the sake of comparability, the PPA (95% CI) of the IgM, IgG, and combined IgM/IgG test results was also calculated on samples evaluated with both tests (paired testing; Tables 3 and 4 and Table S6).

**TABLE 2:** Demographics of French patients positive for SARS-CoV-2 used for the determination of Vidas SARS-CoV-2 IgM and IgG sensitivity (positive percent agreement with RT-PCR positivity)

Vidas SARS-CoV-2 serology testing	IgM	IgG	Combined IgM/IgG
Patients with documented date of RT-PCR-positive test			
No. of samples tested	234	253	232
Study population, *n* (%)	132 (100)	139 (100)	130 (100)
Age in yrs, median (range)			
Nonhospitalized (*n* = 61)	Missing	Missing	Missing
Other (*n* = 71/78/69)	71 (27–96)	70.5 (27–96)	70 (27–96)
Gender, *n* (%)			
Male	47 (35.6)	53 (38.1)	47 (36.2)
Female	24 (18.2)	25 (18.0)	22 (16.9)
Missing	61 (46.2)	61 (43.9)	61 (46.9)
Disease severity, *n* (%)			
Hospitalized	56 (42.4)	57 (41.0)	54 (41.55)
Nonhospitalized	61 (46.2)	61 (43.9)	61 (46.9)
Missing	15 (11.4)	21 (15.1)	15 (11.55)
Patients with documented date of symptom onset			
No. of samples tested	108	113	105
Study population, *n* (%)	65 (100)	71 (100)	63 (100)
Age in yrs, median (range)	70 (27–96)	70 (27–96)	70 (27–96)
Gender, *n* (%)			
Male	45 (69.2)	51 (71.8)	45 (71.4)
Female	20 (30.8)	20 (28.2)	18 (28.6)
Disease severity, *n* (%)			
Hospitalized	50 (76.9)	50 (70.4)	48 (76.2)
Nonhospitalized^*a*^	0 (0.0)	0 (0.0)	0 (0.0)
Missing	15 (23.1)	21 (29.6)	15 (23.8)

a Date of symptom onset not documented in nonhospitalized COVID-19 patients.

**TABLE 3:** Positive percent agreement (PPA) of the Vidas SARS-CoV-2 IgM, IgG and combined IgM/IgG test results of SARS-CoV-2-positive samples according to the time from RT-PCR-positive result

Vidas SARS-CoV-2 serology testing	Time from RT-PCR-positive result (days)	Median time in days (range)	No. of samples^*a*^	No. of positive results	PPA (%)	95% CI (%)
IgM (*n* = 232)	0–7	2 (0–7)	110	49	44.5	35.6–53.9
	8–15	14 (8–15)	60	49	81.7	70.1–89.4
	16–23	20 (16–23)	38	31	81.6	66.6–90.8
	24–31	26 (24–28)	13	13	100.0	75.3–100.0
	≥32	33 (32–65)	11	9	81.8	52.3–94.9
IgG (*n* = 232)	0–7	2 (0–7)	110	50	45.5	36.5–54.8
	8–15	14 (8–15)	60	53	88.3	77.8–94.2
	16–23	20 (16–23)	38	36	94.7	82.7–98.5
	24–31	26 (24–28)	13	13	100.0	75.3–100.0
	≥32	33 (32–65)	11	11	100.0	71.5–100.0
Combined IgM/IgG^*b*^ (*n* = 232)	0–7	2 (0–7)	110	59	53.6	44.4–62.7
	8–15	14 (8–15)	60	57	95.0	86.1–99.0
	16–23	20 (16–23)	38	38	100.0	90.7–100.0
	24–31	26 (24–28)	13	13	100.0	75.3–100.0
	≥32	33 (32–65)	11	11	100.0	71.5–100.0

a No more than one test result per patient per time period.

b Combined test is positive when at least one of the IgM and/or IgG tests is positive.

**TABLE 4:** Positive percent agreement (PPA) of the Vidas SARS-CoV-2 IgM, IgG, and combined IgM/IgG test results of SARS-CoV-2-positive samples according to the time from symptom onset

Vidas SARS-CoV-2 serology testing	Time from symptom onset (days)	Median time in days (range)	No. of samples^*a*^	No. of positive results	PPA (%)	95% CI (%)
IgM (*n* = 105)	0–7	5.5 (1–7)	22	7	31.8	16.4–52.7
	8–15	12 (8–15)	29	24	82.8	65.5–92.4
	16–23	18 (16–23)	26	26	100.0	86.8–100.0
	24–31	26 (24–30)	18	18	100.0	81.5–100.0
	≥32	35 (32–65)	10	10	100.0	69.2–100.0
IgG (*n* = 105)	0–7	5.5 (1–7)	22	7	31.8	16.4–52.7
	8–15	12 (8–15)	29	25	86.2	69.4–94.5
	16–23	18 (16–23)	26	25	96.2	80.4–99.9
	24–31	26 (24–30)	18	17	94.4	74.2–99.0
	≥32	35 (32–65)	10	10	100.0	69.2–100.0
Combined IgM/IgG^*b*^ (*n* = 105)	0–7	5.5 (1–7)	22	8	36.4	19.7–57.0
	8–15	12 (8–15)	29	26	89.7	73.6–96.4
	16–23	18 (16–23)	26	26	100.0	86.8–100.0
	24–31	26 (24–30)	18	18	100.0	81.5–100.0
	≥32	35 (32–65)	10	10	100.0	69.2–100.0

a No more than one test result per patient per time period.

b Combined test is positive when at least one of the IgM and/or IgG tests is positive.

The PPA from the time of RT-PCR positive test results for the Vidas SARS-CoV-2 IgM assay increased from 44.5% at 0 to 7 days to 100.0% at 24 to 31 days before decreasing to 81.8% at ≥32 days (Table 3). The PPA for the Vidas SARS-CoV-2 IgG assay increased from 45.5% at 0 to 7 days to 100.0% from day 24 onward (Table 3). The PPA of the combined IgM and IgG serology tests (positive in at least one of the IgM and/or IgG assays) from the time of RT-PCR positive test results increased from 53.6% at 0 to 7 days to 100.0% from day 16 onward (Table 3 and Table S6). The PPA evaluated relative to the time of symptom onset increased from 31.8% at 0 to 7 days (Vidas SARS-CoV-2 IgM and IgG) to 100.0% from day 16 (Vidas SARS-CoV-2 IgM) or day ≥32 (Vidas SARS-CoV-2 IgG) (Table 4). The PPA of the combined IgM and IgG serology test results from the time of symptom onset increased from 36.4% at 0 to 7 days to 100.0% from day 16 onward (Table 4 and Table S6). It should be noted that the PPA relative to the date of symptom onset is mainly based on hospitalized patients (Table 2).

Based on the specificity and the PPA determined on paired IgM and IgG testing (*n* = 308 for specificity; *n* = 105 for PPA), the negative predictive value (NPV) and the positive predictive value (PPV) were calculated at 5% prevalence (22), according to the time after symptom onset (Table 5). The NPV was high for both the Vidas SARS-CoV-2 IgM and IgG assays, whether considered alone or in combination; the NPV was ≥96.5% (lower 95% confidence limits, ≥95.4%) at 0 to 7 days post-symptom onset, and the NPV increased from 99.1% to 100.0% (lower 95% confidence limits, ≥98.0%) from day 8 onward following symptom onset. The PPV of the Vidas SARS-CoV-2 IgG assay was 100% at all time frames considered. Of note, the PPV calculated using the full data set for the Vidas SARS-CoV-2 IgG specificity determination (*n* = 989) was slightly lower, increasing from 94.3% at 0 to 7 days to 98.1% at ≥32 days (Table S7). The PPV of the Vidas SARS-CoV-2 IgM assay was lower, ranging from 72.1% at 0 to 7 days to 89.0% from day 16 onward following symptom onset (Table 5). The combination of IgM and IgG test results slightly improved the PPV and NPV of the SARS-CoV-2 IgM assay and the NPV of the SARS-CoV-2 IgG assay. The SARS-CoV-2 IgG assay performed best alone in terms of PPV (Table 5).

**TABLE 5:** Positive and negative predictive values (PPV/NPV) at 5% prevalence of the Vidas SARS-CoV-2 IgM, IgG and combined IgM/IgG test results according to the time from symptom onset

Vidas SARS-CoV-2 serology testing	Time from symptom onset	PPV^*a*^^,^^*b*^ (%) (95% CI [%])	NPV^*a*^^,^^*b*^ (%) (95% CI [%])
IgM	0–7	72.1 (36.3–92.1)	96.5 (95.4–97.4)
	8–15	87.0 (62.5–96.4)	99.1 (98.0–99.6)
	16–23	89.0 (67.1–97.0)	100.0 (NA)
	24–31	89.0 (67.1–97.0)	100.0 (NA)
	≥32	89.0 (67.1–97.0)	100.0 (NA)
IgG	0–7	100.0 (NA)	96.5 (95.4–97.4)
	8–15	100.0 (NA)	99.3 (98.2–99.7)
	16–23	100.0 (NA)	99.8 (98.6–100.0)
	24–31	100.0 (NA)	99.7 (98.1–100.0)
	≥32	100.0 (NA)	100.0 (NA)
Combined IgM/IgG	0–7	74.7 (40–92.9)	96.7 (95.6–97.6)
	8–15	87.9 (64.5–96.7)	99.5 (98.4–99.8)
	16–23	89.0 (67.1–97.0)	100.0 (NA)
	24–31	89.0 (67.1–97.0)	100.0 (NA)
	≥32	89.0 (67.1–97.0)	100.0 (NA)

a PPV and NPV were calculated at 5% prevalence and using values of specificity and sensitivity (PPA) determined on paired Vidas SARS-CoV-2 IgM and IgG test results (*n* = 308 for specificity, Table S3; *n* = 105 for PPA, Table 4).

b 95% NA, CI not calculable (division by zero).

### Longitudinal study of IgM and IgG seroconversion in hospitalized COVID-19 patients.

**FIG 2:**
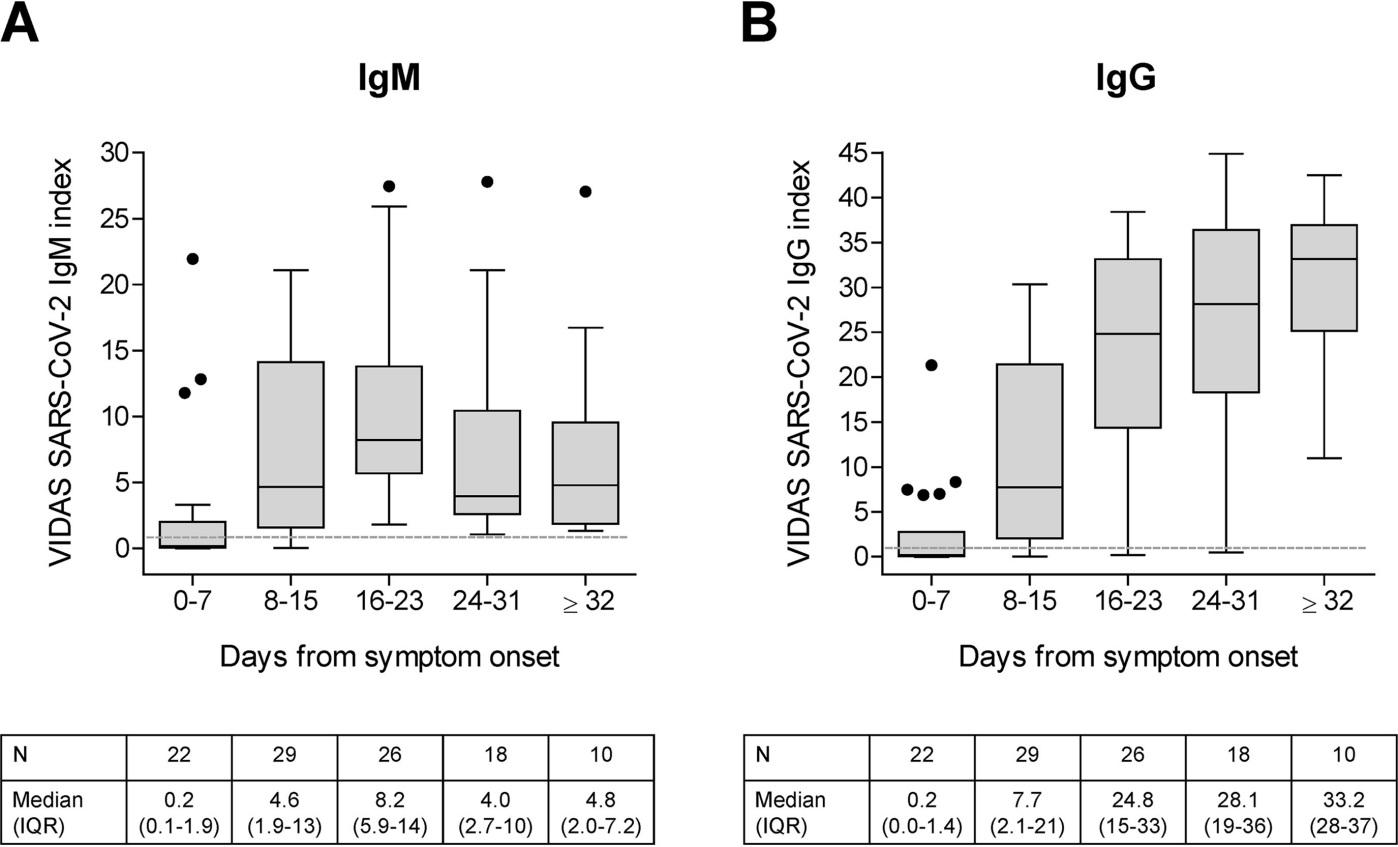
(A and B) Distribution of IgM (A) and IgG (B) index values obtained using the Vidas SARS-CoV-2 IgM and IgG assays, respectively, in patients confirmed positive for SARS-CoV-2, according to the time from symptom onset. Vidas SARS-CoV-2 IgM and IgG index values of 105 paired tests from 63 SARS-CoV-2-positive patients (Table 4, Table 2, and Fig. 1) are displayed as Tukey box plots according to the time from symptom onset. No more than one patient’s sample is included per time frame. The number of tested samples (N), and the median and interquartile range (IQR) of index values are indicated below each graph. The dashed line shows the positivity cutoff of both assays (*i* = 1.00).

The global distribution of Vidas SARS-CoV-2 IgM and IgG index values post-symptom onset was compared among the 105 SARS-CoV-2-positive patients described in Table 4 (i.e., mainly including hospitalized COVID-19 patients; Table 2) (Fig. 2). IgM index values increased from the second week of symptom onset (8 to 15 days) and peaked during the third week (16 to 23 days) before decreasing. In comparison, the IgG index values strongly increased from the second week of symptom onset and seemed to reach a plateau ≥32 days post-symptom onset (Fig. 2).

**FIG 3:**
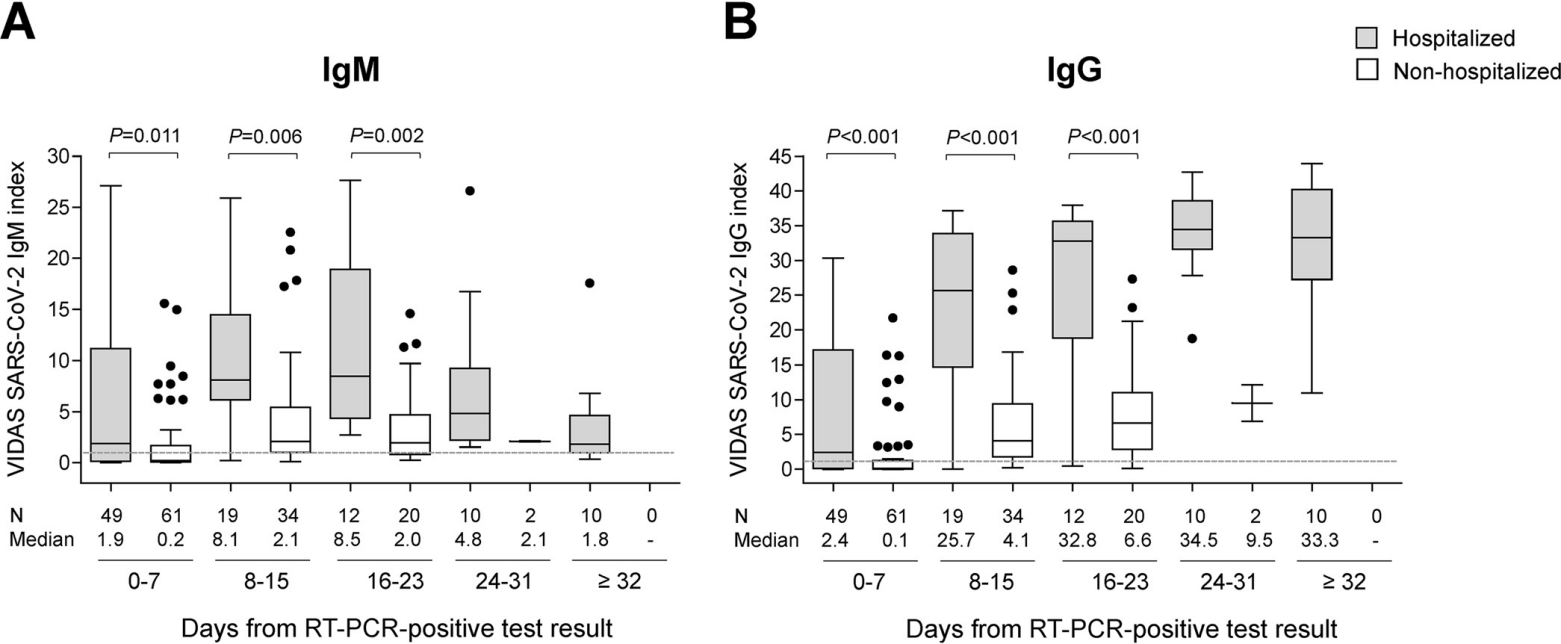
(A and B) Distribution of IgM (A) and IgG (B) index values obtained using the Vidas SARS-CoV-2 IgM and IgG assays, respectively, in hospitalized versus nonhospitalized patients confirmed positive for SARS-CoV-2, according to the time from PCR-positive result. Out of the 232 paired tests (Table 3), 15 were from patients with an unknown hospitalization status (Table 2) and were thus excluded from the analysis. Vidas SARS-CoV-2 IgM and IgG index values of 217 paired tests from 115 SARS-CoV-2-positive patients (100 samples from 54 hospitalized patients and 117 samples from 61 nonhospitalized patients) are depicted as Tukey box plots according to the time from RT-PCR positive test result. No more than one patient’s sample is included per time frame. The number of tested samples (N) and the median index are indicated below each graph. The dashed line shows the positivity cutoff of both assays (*i* = 1.00). Differences between the groups of hospitalized and nonhospitalized patients were tested at each time frame (0 to 7, 8 to 15, and 16 to 23 days post-positive PCR) using a two-sided MWU test; the respective *P* values are displayed above each graph. No statistical testing was performed at 24 to 31 and ≥32 days due to the too low *N* values, notably in the group of nonhospitalized patients.

The Vidas SARS-CoV-2 IgM and IgG index values were also compared between hospitalized and nonhospitalized patients. Since the date of symptom onset was not documented in nonhospitalized patients (Table 2), the index values in 54 hospitalized and 61 nonhospitalized patients were compared relative to the time of the RT-PCR positive results (Fig. 3). Interestingly, the distribution of index values between hospitalized and nonhospitalized patients differs statistically from each other for both the Vidas SARS-CoV-2 IgM and IgG assays at the three compared time frames (0 to 7, 8 to 15, and 16 to 23 days post-PCR positive test; MWU test *P* values < 0.017). Median index values were higher in hospitalized versus nonhospitalized patients (Fig. 3).

**FIG 4:**
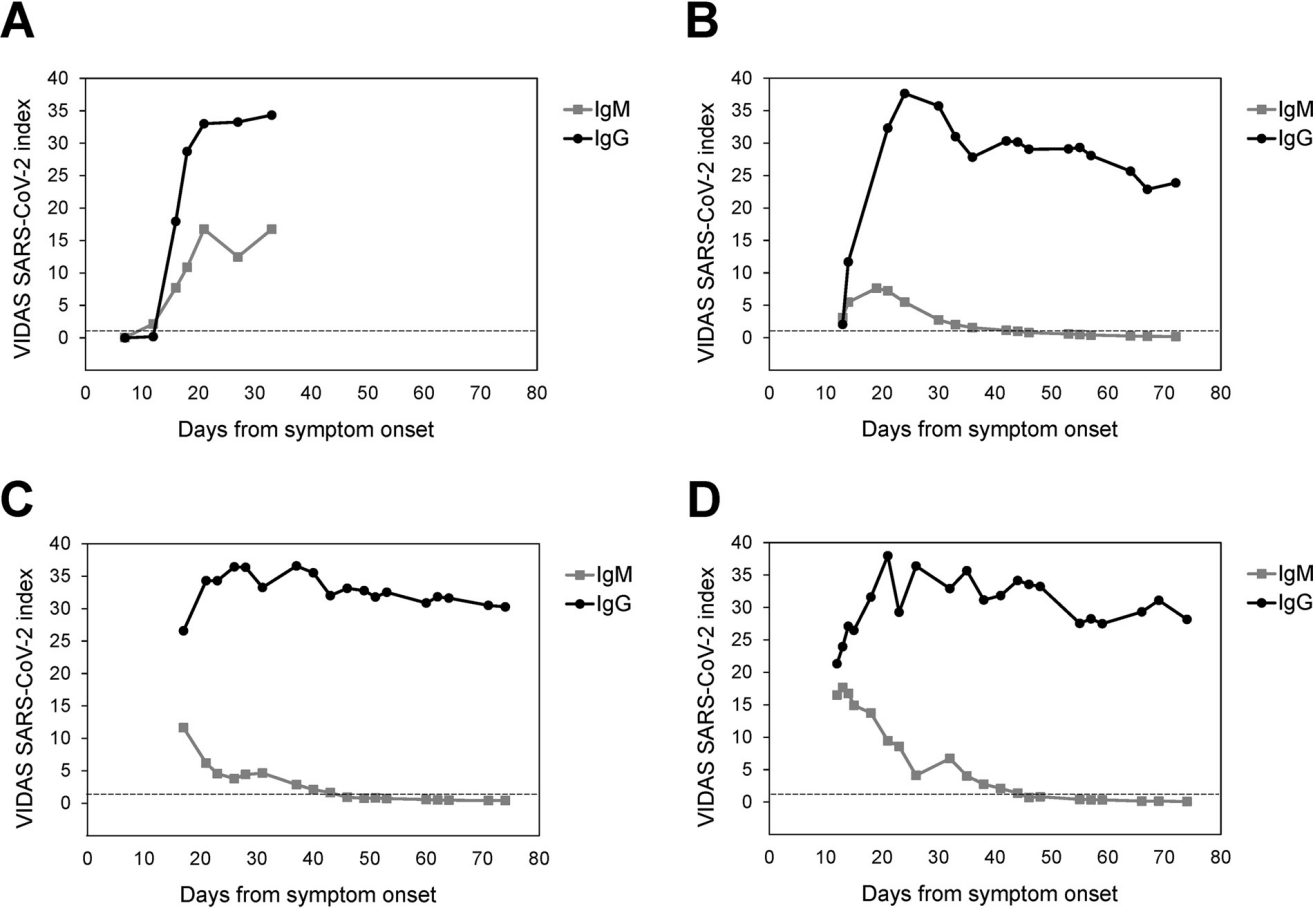
Kinetics of IgM and IgG seroconversion in four selected hospitalized patients. Vidas SARS-CoV-2 IgM and IgG index values of four patients (panels A to D, respectively) measured over time after symptom onset are presented. The dashed line indicates the positivity cutoff of both assays (*i* = 1.00). (A to D) Additional patient information is as follows: (A) the 78-year-old male patient was in the intensive-care unit (ICU) at all investigated time points, except at the first (day 7) and last (day 33) measurement time points; (B) the 77-year-old male patient was in the ICU at all investigated time points; (C) the 43-year-old male patient was in the ICU at all investigated times, except at the last two measurement time points (day 71 and 74); (D) the 67-year-old male patient was in the ICU at all investigated time points.

Finally, IgM and IgG seroconversion were further investigated in four selected hospitalized patients with either early and/or repeated measurements over an extended period of time (up to 74 days post-symptom onset; Fig. 4). IgG seroconversion closely followed IgM seroconversion in the second week of symptom onset (Fig. 4A and B), in line with the global profile shown in Fig. 2. The SARS-CoV-2 IgM index rapidly decreased concomitantly with the increase of the SARS-CoV-2 IgG index (Fig. 4B to D). In the three patients shown in Fig. 4B to D, the SARS-CoV-2 IgM index decreased below the positivity cutoff (index = 1.00) 46 days after symptom onset. In contrast, the SARS-CoV-2 IgG index values remained high and stable from approximately day 20 onward after symptom onset, at least up to 74 days.

## DISCUSSION

We describe here the analytical and clinical performance of the Vidas SARS-CoV-2 IgM and IgG assays. We demonstrate that both assays show high precision and excellent analytical and clinical performance.

The rate of cross-reactivity with nonspecific antibodies, including those of patients infected with other coronaviruses, was very low in both the Vidas SARS-CoV-2 IgM and IgG assays. This weak cross-reactivity with other coronaviruses antibodies is likely due, at least in part, to the choice of the receptor-binding domain (RBD) of the viral spike protein as the SARS-CoV-2-specific antigen. The RBD shows a high sensitivity in enzyme-linked immunosorbent assays (ELISA), higher than that of the SARS-CoV-2 S1 or NC antigens (30–33). It also presents a weaker homology and significant structural divergences with the RBD of other coronaviruses (34–36). Another advantage of using the RBD is that the viral antigen generates neutralizing antibodies likely to provide protective immunity (35–42), as previously demonstrated for SARS-CoV (43, 44). That the Vidas SARS-CoV-2 assays have the ability to detect SARS-CoV-2 neutralizing antibodies was recently demonstrated in mild COVID-19 patients, with an almost perfect concordance (Cohen’s Kappa coefficient of 0.9) between the Vidas SARS-CoV-2 IgG assay and a virus neutralization test (42). Beside its strong immunogenicity and antigenicity, the RBD of SARS-CoV has been shown to elicit antibody responses that persisted many years after infection (38, 44), raising the possibility that it might also be the case for the RBD of SARS-CoV-2. Recent studies in COVID-19 patients, notably in convalescent donors (45–47), on the anti-RBD antibody dynamics post-SARS-CoV-2 infection (39, 40), or demonstrating the persistence and expansion of SARS-CoV-2-specific memory lymphocytes (48), as well as the stability of the IgG response detected with the Vidas SARS-CoV-2 IgG assay up to 74 days post-symptom onset in the present study, strongly support this proposition. Hence, a serology test such as the Vidas SARS-CoV-2 IgG assay is likely to be suitable for the detection of protective immunity and the evaluation of the efficacy of future vaccines, which are mainly based on the RBD-containing spike protein (49, 50).

The low cross-reactivity rate with nonspecific sera probably explains the very high specificity (≥99%) and narrow 95% CI of both the Vidas SARS-CoV-2 IgM and IgG assays. The Vidas SARS-CoV-2 IgG assay alone had a specificity of close to 100%, slightly higher than that of the Vidas SARS-CoV-2 IgM assay.

The clinical sensitivity of the Vidas SARS-CoV-2 assays was evaluated in SARS-CoV-2-confirmed symptomatic cases and was determined as positive percent agreement (PPA) with the RT-PCR assay at successive time frames post-positive PCR and, alternatively, post-symptom onset. The PPA reached 100% at 16 to 23 days (Vidas SARS-CoV-2 IgM) and at ≥32 days (Vidas SARS-CoV-2 IgG) post-symptom onset. The combined Vidas SARS-CoV-2 IgM/IgG test evaluation improved the PPA of the respective IgM and IgG tests by 3.5 to 6.9% during the first 2 weeks (0 to 7 and 8 to 15 days) of symptom onset. Such improved sensitivity of the combined IgM/IgG tests early after symptom onset might also be useful for the diagnosis of suspected COVID-19 cases with negative PCR (13–15, 17, 18).

Overall, the clinical performance of the Vidas SARS-CoV-2 assays was excellent. It was in a range comparable to that reported for existing EUA serological assays (22, 33, 42, 51–57). Moreover, in several side-by-side comparisons of six to nine commercial serological assays (specific for SARS-CoV-2 IgA, IgM, IgG, or total antibodies), Vidas SARS-CoV-2 IgG outperformed some of the IgG-specific competitor assays in terms of specificity and/or PPA with PCR positivity (42, 56, 57). The high specificity of the Vidas SARS-CoV-2 IgG assay alone should be well suited for epidemiological surveillance.

The kinetics of SARS-CoV-2 IgM and IgG seroconversion was also evaluated by monitoring Vidas index values over time. Vidas SARS-CoV-2 IgM and IgG index values increased in the second week after symptom onset. IgG index values strongly increased and remained high, as IgM index values rapidly declined. These profiles are in agreement with those described in recent publications (13, 30, 39, 40, 58–62). Interestingly, the magnitude of the antibody response (index values) correlated with disease severity, as it was significantly higher in hospitalized versus nonhospitalized COVID-19 patients at the time frames investigated (0 to 7, 18 to 15, and 16 to 23 days after a PCR positive test). This observation is in agreement with published reports (11, 40, 62–65). It should be noted that despite the significantly lower response of sera from mild (nonhospitalized) COVID-19 patients in the Vidas SARS-CoV-2 assays (Fig. 3), the good performance of the assays as well as their strong concordance with seroneutralization was recently demonstrated in a cohort of mild COVID-19 patients (42).

This study presents several limitations. First, assay sensitivity was evaluated on confirmed but not on suspected SARS-CoV-2 cases (i.e., patients with symptoms but negative by PCR). It would be interesting to evaluate and confirm the benefit of SARS-CoV-2 IgM and IgG serology to complement PCR testing (13, 14, 17, 18). On the other hand, recent reports suggested that the identification rate of false-negative PCR results using serology testing might be marginal, between ∼1% (20) and ∼4% (62). Second, assay sensitivity was determined on symptomatic (hospitalized and nonhospitalized) COVID-19 patients. The sensitivity of the Vidas SARS-CoV-2 IgM and IgG assays in asymptomatic SARS-CoV-2-infected individuals, who may represent most of the infected patients, remains to be evaluated.

In conclusion, Vidas SARS-CoV-2 IgM and IgG are highly sensitive and specific assays for the reliable screening of patients after acute SARS-CoV-2 infections (and likely after vaccination, when available). Moreover, the Vidas SARS-CoV-2 IgG assay fulfils the specificity requirement for its use in seroepidemiology studies and is well suited for the detection of past SARS-CoV-2 infections. Further studies are necessary to confirm its suitability for the detection of SARS-CoV-2 neutralizing antibodies and to define correlates of immune protection.

## Supplementary Material

Supplemental file 1
