# Supplementary material for: Performance Characteristics of the Vidas SARS-CoV-2 IgM and IgG Serological Assays
Source: J Clin Microbiol. 2021 Mar 19;59(4):e02292-20. doi: 10.1128/JCM.02292-20 (PMC8092742; doi:10.1128/JCM.02292-20)
Supplement: Supplemental file 1 [file JCM.02292-20-s0001.pdf]

Supplemental Material

Table S1. Precision of the VIDAS® SARS-CoV-2 IgM and IgG assays

VIDAS® SARS-CoV-2 assay	Sample	Total number of measurements	Mean index	Repeatability (within-run precision)		Within-laboratory precision	
				SD	CV (%)	SD	CV (%)
IgM	A (negative, high)	60	0.95	0.08	8.5	0.09	9.4
	B (positive)	60	1.50	0.14	9.3	0.16	10.7
	C (positive)	60	5.78	0.42	7.3	0.46	8.0
IgG	D (negative, high)	60	0.88	0.03	3.8	0.04	4.5
	E (positive)	60	1.42	0.08	5.5	0.09	6.5
	F (positive)	60	8.04	0.48	5.9	0.55	6.9

Abbreviations: SD, standard deviation; CV, coefficient of variation

Table S2. Demographics of pre-pandemic healthy volunteers used for the determination of VIDAS® SARS-CoV-2 IgM and IgG specificity

VIDAS® SARS-CoV-2 serology testing	IgM	IgG	Combined IgM/IgG
Study population, N (%)	308 (100%)	989 (100%)	308 (100%)
Age in years, median (range)	45 (18-86)	44 (18-86)	45 (18-86)
Gender, N (%)			
Male	143 (46.4%)	347 (35.1%)	143 (46.4%)
Female	165 (53.6%)	642 (64.9%)	165 (53.6%)
Pre-pandemic healthy volunteers, N (%)			
France	161 (52.3%)	842 (85.1%)	161 (52.3%)
United States	147 (47.7%)	147 (14.9%)	147 (47.7%)

Table S3. Specificity of the VIDAS® SARS-CoV-2 IgM and IgG assays

VIDAS® SARS-CoV-2 serology testing	No. samples	No. negative results	Specificity	95% confidence interval (95% CI)
**IgM**	308	306	99.4%	97.7-99.9%
**IgG**[a]	989	988	99.9%	99.4-100.0%
**Combined IgM/IgG**[b]	308	306	99.4%	97.7-99.9%

[a]Specificity (95% CI) determined based on common samples (N=308) was 100.0% (98.8-100.0%); [b]combined test is negative when both IgM and IgG tests are negative

Table S4. Positive percent agreement (PPA) of the VIDAS® SARS-CoV-2 IgM and IgG test results according to the time from RT-PCR-positive test result (all available samples)

VIDAS® SARS-CoV-2 serology testing	Time from RT-PCR-positive result	Median (range) time in days	No. samples[a]	No. positive results	PPA	95% CI
IgM (N=234)[b]	0-7 days	1.5 (0-7)	112	51	45.5%	36.6-54.8%
	8-15 days	14 (8-15)	60	49	81.7%	70.1-89.4%
	16-23 days	20 (16-23)	38	31	81.6%	66.6-90.8%
	24-31 days	26 (24-28)	13	13	100.0%	75.3-100.0%
	≥ 32 days	33 (32-65)	11	9	81.8%	52.3-94.9%
IgG (N=253)[b]	0-7 days	1 (0-7)	115	47	40.9%	32.3-50.0%
	8-15 days	14 (8-15)	73	63	86.3%	76.6-92.4%
	16-23 days	20 (16-23)	41	39	95.1%	83.5-99.4%
	24-31 days	26 (24-28)	13	13	100.0%	75.3-100.0%
	≥ 32 days	33 (32-65)	11	11	100.0%	71.5-100.0%

[a]no more than one test result per patient per time period; [b]PPA (95% CI) for common samples (N=232) are shown in Table 4

Table S5. Positive percent agreement (PPA) of the VIDAS® SARS-CoV-2 IgM and IgG test results according to the time from symptom onset (all available samples)

VIDAS® SARS-CoV-2 serology testing	Time from symptom onset	Median (range) time in days	No. samples[a]	No. positive results	PPA	95% CI
IgM (N=108)[b]	0-7 days	6 (1-7)	24	9	37.5%	21.2-57.3%
	8-15 days	12 (8-15)	29	24	82.8%	65.5-92.4%
	16-23 days	18 (16-23)	26	26	100.0%	86.8-100.0%
	24-31 days	26 (24-30)	18	18	100.0%	81.5-100.0%
	≥ 32 days	34 (32-65)	11	11	100.0%	71.5-100.0%
IgG (N=113)[b]	0-7 days	5.5 (1-7)	24	9	37.5%	21.2-57.3%
	8-15 days	12 (8-15)	31	25	80.6%	63.7-90.8%
	16-23 days	18 (16-23)	29	28	96.6%	82.2-99.9%
	24-31 days	26 (24-30)	19	18	94.7%	75.4-99.1%
	≥ 32 days	35 (32-65)	10	10	100.0%	69.2-100.0%

[a]no more than one test result per patient per time period; [b]PPA (95% CI) for common samples (N=105) are shown in Table 5

Table S6. Positive percent agreement (PPA) of the VIDAS® SARS-CoV-2 combined IgM/IgG test results and respective number of IgM and IgG positive test results of SARS-CoV-2-positive samples, according to the time from RT-PCR-positive result and of symptom onset

VIDAS® SARS-CoV-2 serology testing	Time window in days[a]	Median (range) time in days[a]	No. samples[b]	No. positive results[c]	VIDAS® IgM positive & IgG negative	VIDAS® IgM positive & IgG positive	VIDAS® IgM negative & IgG positive	PPA	95% CI
**Results relative to documented date of RT-PCR-positive test**									
**Combined IgM/IgG[c] (N=232)**	0-7	2 (0-7)	110	59	9	40	10	53.6%	44.4-62.7%
	8-15	14 (8-15)	60	57	4	45	8	95.0%	86.1-99.0%
	16-23	20 (16-23)	38	38	2	29	7	100.0%	90.7-100.0%
	24-31	26 (24-28)	13	13	0	13	0	100.0%	75.3-100.0%
	≥ 32	33 (32-65)	11	11	0	9	2	100.0%	71.5-100.0%
**Results relative to documented date of symptom onset**									
**Combined IgM/IgG[c] (N=105)**	0-7	5.5 (1-7)	22	8	1	6	1	36.4%	19.7-57.0%
	8-15	12 (8-15)	29	26	1	23	2	89.7%	73.6-96.4%
	16-23	18 (16-23)	26	26	1	25	0	100.0%	86.8-100.0%
	24-31	26 (24-30)	18	18	1	17	0	100.0%	81.5-100.0%
	≥ 32	35 (32-65)	10	10	0	10	0	100.0%	69.2-100.0%

[a]Time from RT-PCR-positive test result (upper Table) or from symptom onset (lower Table); [b]no more than one patient's test result per time period; [c]combined test is positive when at least one of IgM and/or IgG tests are positive

Table S7. Positive and negative predictive values (PPV/NPV) at 5% prevalence of the VIDAS® SARS-CoV-2 IgM, IgG and combined IgM/IgG test results, according to the time from symptom onset (all available samples for IgG specificity)

VIDAS® SARS-CoV-2 serology testing	Time from symptom onset	PPV[a] (95% CI)	NPV[a] (95% CI)
**IgM**	0-7 days	72.1% (36.3-92.1%)	96.5% (95.4-97.4%)
	8-15 days	87.0% (62.5-96.4%)	99.1% (98.0-99.6%)
	16-23 days	89.0% (67.1-97.0%)	100.0% (N/A)[b]
	24-31 days	89.0% (67.1-97.0%)	100.0% (N/A)[b]
	≥ 32 days	89.0% (67.1-97.0%)	100.0% (N/A)[b]
**IgG**	0-7 days	94.3% (68-99.2%)	96.5% (95.4-97.4%)
	8-15 days	97.8% (86.3-99.7%)	99.3% (98.2-99.7%)
	16-23 days	98.0% (87.6-99.7%)	99.8% (98.6-100.0%)
	24-31 days	98.0% (87.4-99.7%)	99.7% (98.1-100.0%)
	≥ 32 days	98.1% (88-99.7%)	100.0% (N/A)[b]
**Combined IgM/IgG**	0-7 days	74.7% (40-92.9%)	96.7% (95.6-97.6%)
	8-15 days	87.9% (64.5-96.7%)	99.5% (98.4-99.8%)
	16-23 days	89.0% (67.1-97.0%)	100.0% (N/A)[b]
	24-31 days	89.0% (67.1-97.0%)	100.0% (N/A)[b]
	≥ 32 days	89.0% (67.1-97.0%)	100.0% (N/A)[b]

[a]PPV and NPV were calculated at 5% prevalence using values of sensitivity (PPA) determined on paired IgM and IgG test results (N=105; Table 5); values of specificity were those calculated on all available samples (N=308 for IgM and N=989 for IgG; Table 3); [b]95% CI not calculable (division by zero)
